# Clinical features, multi-organ damage, and prognosis of 1,1-dichloroethylene poisoning: a retrospective case series of 13 patients

**DOI:** 10.3389/fpubh.2026.1869600

**Published:** 2026-06-22

**Authors:** Yongxin Sun, Chunyan Tian, Xiang Gao, Zhixin Ji, Xin Sun, Liming Wang, Qinghai Zhang

**Affiliations:** 1 Department of Critical Care Medicine, Weifang People's Hospital, Shandong Second Medical University, Weifang, China; 2 Department of Health Comprehensive Geriatrics, Weifang Yidu Central Hospital, Weifang, China

**Keywords:** 11-dichloroethylene, acute kidney injury, hepatic insufficiency, occupational exposure, toxic actions

## Abstract

**Background:**

1,1-Dichloroethylene (1,1-DCE) is a widely used chlorinated solvent with known hepatotoxicity in animals, but human acute poisoning data are scarce. This study summarizes clinical features and multi-organ injury in acute 1,1-DCE poisoning.

**Methods:**

A retrospective case series of 13 patients with single occupational exposure was conducted. Clinical data, laboratory parameters (liver, renal, coagulation, hematologic, and cardiac markers), organ support, and outcomes were analyzed.

**Results:**

All patients developed early symptoms. Severe hepatocellular injury was prominent (median alanine aminotransferase 3,400 U/L; aspartate aminotransferase 2,516 U/L). Five patients (38.5%) developed acute kidney injury, including four with anuria. All showed coagulation abnormalities with elevated D-dimer, and 12 had myocardial injury. Organ support included mechanical ventilation (30.8%), continuous renal replacement therapy (61.5%), and veno-venous extracorporeal membrane oxygenation (7.7%). Three patients (23.1%) died of multiple organ failure.

**Conclusion:**

Acute 1,1-DCE exposure rapidly causes multi-organ dysfunction, dominated by severe liver injury with renal, coagulation, and cardiac involvement. Early monitoring and aggressive supportive treatment are critical.

## 1 Introduction

The 1,1-DCE is a volatile chlorinated alkene widely used in the production of polyvinylidene chloride (PVDC) resins and related coating materials. Under routine industrial conditions, exposure levels are generally low and are unlikely to result in overt toxicity. However, in confined spaces or in the event of accidental release due to operational errors, high-concentration exposure may occur within a short period of time, leading to severe acute toxicity (1).

Experimental studies have demonstrated that 1,1-DCE undergoes hepatic metabolism primarily via cytochrome P450 2E1 (CYP2E1), generating highly reactive intermediates that can induce hepatocellular necrosis, renal tubular injury, and widespread oxidative stress. Multiple animal models, including rats and mice, have consistently shown that 1,1-DCE exposure can result in multi-organ injury involving the liver, kidneys, and lungs (1, 2). However, systematic evidence regarding the clinical manifestations and patterns of organ involvement following acute 1,1-DCE exposure in humans remains limited.

A recent occupational exposure incident resulted in the simultaneous hospitalization of 13 exposed individuals, forming a well-defined cohort of patients with acute 1,1-DCE poisoning characterized by a common exposure source and clearly documented exposure timing. To date, no systematic case series focusing on acute 1,1-DCE poisoning has been reported. Therefore, this study aims to comprehensively characterize the clinical features, dynamic changes in laboratory parameters, and outcomes of patients with acute 1,1-DCE exposure, to further elucidate the patterns of multi-organ injury and the underlying mechanisms. The findings are expected to provide a valuable basis for the clinical management and prevention of acute 1,1-DCE poisoning in the future.

## 2 Materials and methods

This was a single-center, retrospective case series including 13 patients admitted to our hospital following the same occupational exposure incident.

### 2.1 Exposure assessment and definitions

All patients were involved in a single occupational exposure incident that occurred during cargo unloading operations in an enclosed workspace in the early morning (ambient temperature approximately 22 °C). An accidental release of a volatile chemical substance occurred during handling of the cargo. Following the incident, a police investigation confirmed the source and identity of the transported chemical, and subsequent environmental monitoring identified 1,1-dichloroethylene (1,1-DCE) as the exposure agent. Exposure verification was based on the police investigation findings, environmental monitoring results, and the absence of other known toxic chemical exposures. Environmental monitoring identified airborne concentrations of 1,1-DCE at the worksite. No other major toxic chemicals were identified during the investigation. Exposure timing was determined from occupational incident records and interviews with affected workers. All patients were exposed during the same event, and the interval between exposure and hospital presentation was documented whenever available.

Data collected in this study included patients’ demographic characteristics, exposure circumstances, initial clinical presentations, vital signs, and organ support interventions, as well as laboratory data obtained at admission and throughout hospitalization. These laboratory parameters comprised liver and renal function tests, complete blood counts, coagulation profiles, myocardial injury biomarkers, and inflammatory markers. All laboratory measurements were performed in the same hospital laboratory using standardized clinical assays and institutional quality-control procedures. Therefore, laboratory methodology was consistent across all included patients. Acute kidney injury (AKI) was defined and staged according to the Kidney Disease: Improving Global Outcomes (KDIGO) criteria (3). Acute liver injury was defined as alanine aminotransferase (ALT) ≥ 2 times the upper limit of normal (ULN) (4). Coagulation abnormalities were defined as a prothrombin time (PT) prolongation >3 s, activated partial thromboplastin time (APTT) prolongation >10 s, or an international normalized ratio (INR) outside the reference range.

### 2.2 Statistical analysis

Given the limited sample size, descriptive statistical methods were primarily used to summarize clinical characteristics and laboratory trends. For each patient, the admission value was defined as the first recorded measurement. The maximum value was defined as the highest value recorded during hospitalization, and the time to peak was defined as the number of days from admission to the first occurrence of the maximum value. Similarly, the minimum value was defined as the lowest recorded value during hospitalization, with time to nadir defined as the number of days from admission to its first occurrence. The pre-discharge value was defined as the last available measurement obtained prior to hospital discharge. Anuria was defined as a 24-h urine output <100 mL or complete absence of urine output over 12 h. Cardiac troponin I (cTnI) values exceeding the upper detection limit (>27,404) were recorded as 27,404. All data were expressed as median (interquartile range). Missing data were infrequent and mainly resulted from unavailable tests during routine clinical care. Missing values were not imputed because of the small sample size and the descriptive nature of the study. Analyses were performed using available-case data. All statistical analyses were performed using IBM SPSS Statistics version 27.0.

### 2.3 Ethics statement

This retrospective study involved only the collection of clinical data and routine examination results and posed no additional risk to patients. All patient information was handled with strict confidentiality to protect privacy. As the study utilized identifiable data from previously hospitalized patients and did not involve personal privacy concerns or commercial interests, obtaining informed consent was deemed impracticable. Therefore, the requirement for informed consent was waived. The study protocol was approved by the Ethics Committee of Weifang People’s Hospital (protocol code KYLL20260417-11).

## 3 Results

### 3.1 Baseline characteristics and clinical outcomes

A total of 13 patients with acute 1,1-DCE poisoning were included in this study. The median age was 43 years (IQR 34–54), with two patients aged >65 years. All cases originated from the same occupational exposure incident. Following admission, patients exhibited rapid clinical deterioration, and 12 patients (92.3%) required admission to the intensive care unit (ICU) for close monitoring and organ support. The median length of hospital stay was 21 days (IQR 13–24), while the median ICU stay was 9 days (IQR 4–18). Six patients (46.2%) had both hospital and ICU stays exceeding 10 days. Overall, Acute Physiology and Chronic Health Evaluation II (APACHE II) scores were relatively high (Table 1), indicating substantial physiological derangement at an early stage after admission.

**Table 1:** Patient basic information.

Patient ID	Gender	Age	Die	Clinic time	Length of stay	Length of stay in the ICU	APACHE II	Symptom
1	Man	42	Yes	First day	31	31	25	Nausea, emesis and chest distress
2	Man	72	Yes	First day	32	32	18	Nausea, chest distress, difficult breathing and abdominal discomfort
3	Man	54	No	First day	21	14	13	Nausea, chest distress and disturbance of consciousness
4	Woman	74	No	First day	26	19	22	Nausea, emesis, dizzy and chest distress
5	Man	53	No	First day	24	9	17	Nausea and dizzy
6	Man	33	No	First day	24	9	18	Nausea, emesis and difficult breathing
7	Man	34	No	First day	13	4	11	No obvious discomfort (no direct contact)
8	Man	43	No	First day	13	3	12	Nausea, emesis and abdominal discomfort
9	Man	53	No	First day	13	3	9	Abdominal discomfort
10	Man	33	No	Next day	8	0	-	No obvious discomfort (Detecting abnormal liver function)
11	Man	55	Yes	Four days later	12	12	30	Chest distress
12	Man	34	No	Ten days later	24	9	18	Nausea, emesis, chest distress and difficult breathing
13	Man	37	No	Ten days later	20	18	20	Nausea and emesis

In terms of clinical outcomes, nine patients (69.2%) were transferred from the ICU to the general ward after receiving intensive treatment and subsequently improved to discharge. One patient (7.7%), who had no direct chemical exposure and presented with relatively mild symptoms, did not require ICU admission. Despite aggressive management, three patients (23.1%) died in the context of multiple organ dysfunction (Table 1).

### 3.2 Hepatic injury

Hepatic injury was observed in all 13 patients and was markedly severe (Tables 2, 3). On the first day of admission, the median serum ALT level was 3,400 U/L (IQR 148–4,318), and the median aspartate aminotransferase (AST) level was 2,516 U/L (IQR 135–7,074), indicating substantial hepatocellular damage at presentation (Table 2). Within 48 h after admission (median time to peak: 1 day [IQR 1–2]), ALT and AST levels reached their maximum values, with medians of 3,621 U/L (IQR 244–5,004) and 5,404 U/L (IQR 305–8,756), respectively (Table 3). Total bilirubin (TBIL) had a median value of 26.1 μmol/L (IQR 19.75–34.5) on admission and peaked at a median of 36.9 μmol/L (IQR 25.7–64), with a median time to peak of 4 days (IQR 2–10) (Table 3). These findings suggest that, in addition to hepatocellular necrosis, impaired biliary excretion was also present.

**Table 2:** Patient blood counts, biochemistry, coagulation indices (Admission and discharge, as well as individual extreme values).

Parameter	Admission (day 1)	Number of patients with abnormal indicators	Abnormal indicator (maximum/minimum)	Pre-discharge
RBC (×10^12^/L)	4.38 (3.15–4.49)	9	1.69 (Minimum)	3.42 (3.03–4.56)
WBC (×10^9^/L)	7.94 (6.54–10.31)	12	35.78 (Maximum)	5.61 (3.84–7.19)
PLT (×10^9^/L)	88 (61–129)	3	7 (Minimum)	172 (144–203)
ALT (U/L)	3,400 (148–4,318)	13	5,940 (Maximum)	37 (17–118)
AST (U/L)	2,516 (135–7,074)	13	19,391 (Maximum)	23 (18–34)
TBIL (μmol/L)	26.1 (19.75–34.5)	12	353.3 (Maximum)	15.9 (11.1–26.4)
CREA (μmol/L)	72 (67–186)	5	740 (Maximum)	56 (52–275)
PT (s)	15.7 (13.6–16.1)	11	25.8 (Maximum)	13 (11.5–14.6)
APTT (s)	32.2 (29.9–43.5)	9	156.1 (Maximum)	26.8 (26.4–35.3)
D-dimer (mg/L)	3.155 (0.99–6.32)	12	100.5 (Maximum)	0.87 (0.50–2.97)
INR	1.36 (1.23–1.37)	12	2.28 (Maximum)	1.11 (0.99–1.27)
PALB (mg/L)	277.7 (230.75–294.3)	8	85.66 (Minimum)	195.4 (145.46–235.83)

**Table 3:** Patient blood counts, biochemistry, coagulation indices (during hospitalization).

Parameter	Peak during hospitalization	Time to peak (days)	Nadir during hospitalization	Time to nadir (days)
RBC (×10^12^/L)	4.49 (4.13–5.01)	4 (1–7)	2.60 (2.06–4.12)	6 (2–10)
WBC (×10^9^/L)	17.47 (15.09–20.2)	7 (3–11)	5.16 (3.84–5.82)	19 (2–24)
PLT (×10^9^/L)	180 (144–256)	13 (8–19)	40 (21–89)	1 (1–3)
ALT (U/L)	3,621 (244–5,004)	1 (1–2)	28 (17–54)	14 (13–23)
AST (U/L)	5,404 (305–8,756)	1 (1–2)	22 (14–30)	13 (10–19)
TBIL (μmol/L)	36.9 (25.7–64)	4 (2–10)	15.9 (10.6–20.5)	13 (5–18)
CREA (μmol/L)	76 (67–539)	1 (1–4)	56 (46–117)	6 (3–9)
PT (s)	17.8 (15.9–19.2)	2 (1–3)	11.5 (10.2–12.9)	12 (3–17)
APTT (s)	47.7 (32.2–106.8)	2 (1–3)	26.2 (24.5–27.4)	6 (3–11)
D-dimer (mg/L)	14.06 (7.08–49.25)	4 (3–6.25)	0.775 (0.495–1.39)	10.5 (3.25–18.75)
INR	1.55 (1.36–1.68)	2 (1–3)	0.99 (0.88–1.11)	12 (3–17)
PALB (mg/L)	258.23 (238.45–301.68)	4.5 (1–8.5)	175.015 (142.18–203.57)	5 (3.75–15.25)

Regarding hepatic synthetic function, the median prealbumin (PALB) level on admission was 277.7 mg/L (IQR 230.75–294.3), which further declined during hospitalization, reaching a nadir on day 5 (IQR 4–15) (Table 3). The median lowest PALB level was 175.0 mg/L (IQR 142.2–203.6), indicating a marked impairment of hepatic synthetic capacity (Table 3). With clinical improvement, serum transaminases and bilirubin levels gradually decreased during the later stage of hospitalization; however, 10 patients (76.9%) had not returned to normal ranges by the time of discharge (Table 2). Two patients (15.3%) met the criteria for severe acute liver injury. Combined with other laboratory findings, these findings suggest progression to multiple organ dysfunction syndrome characterized primarily by hepatic failure, requiring organ supportive therapies, including plasma exchange, CRRT, and respiratory support if necessary.

### 3.3 Renal dysfunction

AKI was a prominent clinical feature in this cohort. The median serum creatinine level on admission was 72 μmol/L (IQR 67–186) (Table 2). Notably, within the first 24 h after admission, 5 patients (38.5%) developed a significant rise in serum creatinine, indicating early renal involvement. Furthermore, 4 patients (30.8%) exhibited anuria on day 1 of hospitalization (Table 4), suggesting a severe degree of renal impairment.

**Table 4:** Urine output and CTNI.

Variable	Summary
Urine output	
Median daily urine output, mL (IQR)	3,000 (200–3,600)
Minimum urine output, mL (IQR)	0 (0–1850)
Patients with anuria, *n* (%)	4(30.8%)
Duration of anuria, days, median (IQR)	0 (0–7)
Cardiac injury marker (CTNI)	
Peak CTNI, median (IQR)	136.0 (50.8–383.7)
Exceeding upper detection limit, *n* (%)	1 (7.7%)
Time to peak CTNI, days, median (IQR)	26 (16–30)

Integrating organ support data across the cohort, 8 patients (61.5%) required CRRT (Table 5). Among the 10 survivors, serum creatinine levels and urine output showed gradual improvement during the later stages of hospitalization. However, 2 patients (20%) continued to exhibit persistent renal insufficiency at discharge, classified as stage 3 and stage 4 chronic kidney disease (CKD), respectively. Notably, one of these patients remained anuric, reflecting irreversible or only minimally reversible renal injury.

**Table 5:** Organ support situation.

Patient ID	Respiratory system support measures	Renal support measures	Number of plasma exchange
1	Tracheal intubation + ventilator-assisted breathing; VV-ECMO	CVVH	9
2	Tracheal intubation + ventilator-assisted breathing	CVVH+CVVHDF	5
3	Tracheal intubation + ventilator-assisted breathing	CVVH	4
4	High-flow oxygen inhalation	CVVH	3
5	High-flow oxygen inhalation	CVVH	3
6	High-flow oxygen inhalation	CVVH	NO
7	Nasal cannula oxygen inhalation	NO	NO
8	Nasal cannula oxygen inhalation	NO	NO
9	Nasal cannula oxygen inhalation	NO	NO
10	NO	NO	NO
11	Tracheal intubation + ventilator-assisted breathing	CVVH	11
12	High-flow oxygen inhalation	NO	NO
13	Nasal cannula oxygen inhalation	CVVH\CVVHDF	NO

### 3.4 Coagulation abnormalities and hematological changes

Coagulation abnormalities were observed in 12 patients (Tables 2, 3). On admission, the median PT was 15.7 s (IQR 13.6–16.1), APTT was 32.2 s (IQR 29.9–43.5), and INR was 1.36 (IQR 1.23–1.37), indicating early impairment of coagulation factor synthesis (Table 2). Coagulation parameters further deteriorated the first few days after admission, reaching peak abnormalities within a median of 2 days (IQR 1–3) (Table 3). The median peak PT increased to 17.8 s (IQR 15.9–19.2), consistent with acute injury to the coagulation system (Table 3). D-dimer levels were markedly elevated at presentation, with a median of 3.155 mg/L (IQR 0.993–6.315), and further increased to a median peak of 14.06 mg/L (IQR 7.08–49.25), suggesting significant activation of the coagulation–fibrinolysis system (Table 3). To correct coagulopathy, 10 patients (76.9%) required transfusion of fresh frozen plasma and/or platelets, highlighting the clinical severity and the need for active hemostatic support.

With respect to hematological parameters, the median red blood cell (RBC) count on admission was 4.38 × 10^12^/L (IQR 3.15–4.49), which declined during hospitalization to a median nadir of 2.60 × 10^12^/L (IQR 2.06–4.12) (Tables 2, 3). The median white blood cell (WBC) count on admission was 7.94 × 10^9^/L (IQR 6.54–10.31), and elevated levels were observed in 12 patients (92.3%) (Tables 2, 3). The median platelet count on admission was 88 × 10^9^/L (IQR 61–129), with a nadir median of 40 × 10^9^/L (IQR 21–89) during hospitalization (Tables 2, 3). In two patients, the platelet count reached the lowest value among all 13 cases (7 × 10^9^/L) on the first day of admission, suggesting the presence of platelet consumption and/or suppression (Table 2).

### 3.5 Cardiac biomarkers

Elevations in cardiac injury biomarkers were observed in all patients during hospitalization, suggesting possible myocardial injury. Notably, 4 patients (30.8%) exhibited marked increases in cTnI, with a median level of 136.0 ng/L (IQR 50.8–383.7) (Table 4). One patient demonstrated an extreme elevation exceeding the upper detection limit (>27,404) (Table 4), consistent with significant biomarker elevation.

Abnormalities in myocardial injury biomarkers suggest that acute 1,1-DCE intoxication may trigger a multi-organ response. Notably, myocardial injury does not appear to be solely attributable to hypoxia in all cases but may be related to toxic exposure itself, systemic inflammation, hypoxia, metabolic disturbances, or a combination of these factors.

### 3.6 Organ support and in-hospital outcomes

In terms of respiratory support, 4 patients required endotracheal intubation and invasive mechanical ventilation, while an additional 4 patients received high-flow oxygen therapy (Table 5). Notably, one patient (7.7%) developed refractory respiratory failure and required VV-ECMO on day 7 after admission (Table 5). Renal support was frequently required, with 8 patients undergoing CRRT, underscoring both the high incidence and severity of AKI in this cohort. In addition, 6 patients (46.15%) received plasma exchange therapy at varying frequencies (Table 5). Despite timely and comprehensive supportive management, 3 patients experienced rapid clinical deterioration and ultimately died in the context of progressive multiple organ failure.

Overall, patients requiring plasma exchange and renal replacement therapy tended to exhibit more severe laboratory abnormalities and worse clinical outcomes. Acute 1,1-DCE poisoning was characterized by a distinct pattern of multi-organ injury, with severe hepatic damage as the central feature, accompanied by concurrent involvement of the kidneys, coagulation system, and myocardium. Laboratory abnormalities typically occurred early, were often pronounced, and persisted for a prolonged duration in a subset of patients, reflecting sustained systemic toxicity and ongoing organ dysfunction.

Collectively, these findings indicate that acute 1,1-DCE toxicity follows an aggressive clinical course, frequently necessitating multi-organ support, and highlight the importance of early recognition, close monitoring, and timely initiation of advanced supportive therapies to potentially improve outcomes.

## 4 Discussion

This study represents the largest case series to date of acute 1,1-DCE poisoning following a single, clustered occupational exposure. From the perspectives of multi-organ laboratory findings, supportive therapies, and clinical outcomes, we systematically characterized the toxic effects of 1,1-DCE in humans. Our findings demonstrate that patients with acute 1,1-DCE poisoning can develop severe hepatic injury within a short period after exposure, as reflected by markedly elevated aminotransferase levels on admission (median ALT and AST of 3,400 U/L and 2,516 U/L, respectively). In addition to hepatotoxicity, patients frequently presented with thrombocytopenia, AKI (with some cases progressing to anuria requiring CRRT), coagulation abnormalities (elevated INR and D-dimer), and increased cardiac injury biomarkers. These findings indicate that the toxicity of 1,1-DCE is not confined to a single target organ but instead represents a systemic process involving multi-organ injury.

In the following section, we integrate existing experimental and toxicological evidence to further elucidate the underlying mechanisms and clinical implications of this poisoning syndrome.

### 4.1 Hepatic injury: consistent with prior toxicological evidence but of greater severity

The liver is widely recognized as one of the primary target organs of 1,1-DCE. Previous animal studies, environmental toxicology investigations, and occupational health assessments have consistently demonstrated its hepatotoxic potential (1, 2). In the present case series, patients exhibited markedly elevated aminotransferase levels at the time of admission, indicating that severe hepatic injury had already developed prior to clinical presentation. In addition to marked aminotransferase elevation, abnormalities in bilirubin and PALB suggest impairment of both biliary excretion and hepatic synthetic function. These findings are in line with animal toxicology studies demonstrating that 1,1-DCE can induce hepatocellular necrosis and steatosis (5–8), although the severity of injury observed in the present study appears to be greater.

The hepatotoxicity of 1,1-DCE is primarily attributed to its metabolic activation in the liver via the cytochrome P450 system, particularly CYP2E1. Evidence suggests that 1,1-DCE is biotransformed into reactive intermediates, predominantly DCE epoxide, which can conjugate with glutathione (GSH), representing a key intracellular detoxification pathway. However, when hepatic GSH reserves are depleted, these reactive metabolites that are insufficiently detoxified can enhance protein adduct formation and induce membrane damage in hepatocytes, ultimately leading to centrilobular hepatocellular necrosis and steatosis. Furthermore, long-term animal studies have demonstrated that repeated or chronic exposure to 1,1-DCE results in a spectrum of hepatic pathological changes, ranging from steatosis to inflammation and hepatocellular necrosis. These alterations are often accompanied by hepatomegaly and elevated liver enzyme levels, findings that are consistent with the severe hepatic injury observed in our cohort (6, 7, 9, 10).

Public toxicological reviews and regulatory assessments, including those from the Agency for Toxic Substances and Disease Registry, United States Environmental Protection Agency, and International Programme on Chemical Safety, have reached consistent conclusions regarding the hepatotoxic and nephrotoxic effects of 1,1-DCE. Although robust evidence exists from animal studies, direct clinical data in humans remain limited (1).

In the present study, hepatic injury occurred early—within 24 h of exposure—and was not limited to elevations in aminotransferases. Patients also exhibited impaired synthetic function (e.g., decreased PALB levels) and increased bilirubin concentrations. These findings suggest that, in addition to providing aggressive supportive care, clinicians should monitor not only markers of hepatocellular injury but also remain vigilant for the potential progression to hepatic failure.

### 4.2 High incidence of AKI, indicating significant nephrotoxicity

To date, no case series of 1,1-DCE poisoning have systematically described renal involvement. In the present study, we demonstrate in this case series that acute exposure to 1,1-DCE is associated with a high incidence and rapid progression of AKI. Notably, four patients developed anuria early during hospitalization and required support with CRRT, highlighting the severity of renal impairment.

These clinical findings are consistent with prior experimental evidence. Animal studies have shown that 1,1-DCE can induce significant renal tubular injury, accompanied by elevations in blood urea nitrogen and serum creatinine levels (1, 10, 11). Experimental studies indicate that the kidney is an additional target organ of 1,1-DCE toxicity. Renal tubular epithelial cells may be particularly vulnerable because of local metabolic activation and susceptibility to oxidative injury (12–14). In animal models, characteristic pathological changes have been observed, including degeneration of proximal tubular cells, loss of the brush border, and tubular luminal necrosis. These findings are closely associated with the local formation and accumulation of toxic metabolites within the kidney (10, 13).

Compared with hepatic injury, renal toxicity associated with 1,1-DCE may exhibit distinct characteristics. On the one hand, the kidney functions not only as an excretory organ but also possesses a certain degree of local metabolic capacity, resulting in sustained exposure to high concentrations of toxic metabolites following systemic absorption. On the other hand, renal tubular epithelial cells are highly susceptible to oxidative stress and are therefore more prone to secondary injury in the setting of systemic inflammation, hypoperfusion, or concomitant hepatic dysfunction (14).

Notably, the occurrence of AKI in this case series cannot be fully explained by conventional prerenal factors (e.g., hypotension, shock, or hypoxia). Four patients developed a rapid rise in serum creatinine and oliguria despite relatively stable hemodynamic status, supporting the presence of a direct nephrotoxic effect of 1,1-DCE. Furthermore, the severity of AKI was closely associated with the need for CRRT, suggesting that renal dysfunction is not merely a concomitant manifestation but rather a critical determinant of disease severity and clinical prognosis.

The findings of this study have important implications for clinical practice. In patients with suspected or confirmed acute 1,1-DCE poisoning, renal function should be closely monitored from the early stage of hospital admission, with prompt recognition of the onset of AKI. In critically ill patients, early assessment of indications for renal replacement therapy is warranted to avoid delays in initiating supportive treatment and to optimize clinical outcomes.

### 4.3 Coagulation abnormalities indicate systemic toxicity

In the present study, 12 patients exhibited coagulation abnormalities, as reflected by a median INR of 1.36 and a median D-dimer level of 3.155 mg/L. Additionally, 10 patients required blood transfusion or plasma exchange. While severe hepatic injury can impair the synthesis of coagulation factors and lead to prolongation of PT/INR, the markedly elevated D-dimer levels observed in this cohort suggest activation of the coagulation system. This pattern raises the possibility of consumptive coagulopathy or DIC; however, definitive diagnosis of DIC could not be established because fibrinogen levels, DIC scores, and other confirmatory assessments were not systematically available (15). Importantly, coagulation abnormalities in this study were early in onset and pronounced in severity. Their temporal profile and clinical presentation suggest that they are not merely secondary to hepatic failure but more likely reflect a toxicologically mediated process involving systemic inflammation and endothelial injury (15, 16). In cases where coagulation dysfunction is solely attributable to hepatic failure, the predominant mechanism is reduced synthesis of coagulation factors, typically manifested as decreased levels of clotting factors, reduced fibrinogen, and a bleeding tendency. In contrast, the concurrent elevation of D-dimer in our cohort indicates significant activation of fibrinolysis and increased generation of fibrin degradation products. This pattern may be compatible with DIC or systemic coagulation activation (15, 17).

From a pathophysiological perspective, chemical toxicants—including certain halogenated hydrocarbons and organic solvents—may induce systemic coagulation dysregulation through at least three non-mutually exclusive mechanisms. First, direct endothelial injury or dysfunction plays a central role: reactive metabolites can interact with endothelial cells, inducing tissue factor (TF) expression and exposing procoagulant surfaces, thereby triggering the extrinsic coagulation pathway (18). Second, the release of proinflammatory cytokines—such as tumor necrosis factor-*α* (TNF-α), interleukin IL-1, and IL-6—can further amplify coagulation activation by upregulating TF expression on monocytes and endothelial cells while simultaneously suppressing natural anticoagulant pathways, including the protein C system. This imbalance shifts the hemostatic equilibrium toward a prothrombotic state and promotes microvascular thrombosis (19). Third, products associated with hemolysis or cellular necrosis—such as free hemoglobin, heme, and other intracellular components—can directly activate the vascular endothelium and platelets, thereby amplifying the coagulation–inflammation feedback loop (20). Collectively, these mechanisms have been well documented in toxin-associated coagulopathies and in the pathogenesis of DIC (21).

For the present study, interpreting coagulation abnormalities solely as a passive consequence of impaired hepatic synthetic function is insufficient to fully explain the early onset of coagulation disturbances, the marked elevation of D-dimer levels, and the occurrence of thrombosis or focal microcirculatory alterations observed in a subset of patients (15, 17). A more plausible explanation is that, under conditions of high-dose acute 1,1-DCE exposure, may contribute to endothelial dysfunction. However, this hypothesis cannot be directly confirmed by the present clinical data. Similar patterns of systemic coagulopathy have been reported in other forms of acute chemical intoxication, including heavy metal poisoning and exposure to certain organic solvents, highlighting the “toxicity–inflammation–coagulation” axis as a critical framework for understanding complications in severe poisoning (22, 23).

This study suggests several important clinical implications. First, in patients with suspected acute exposure to 1,1-DCE or other highly toxic organic solvents, early and parallel monitoring of liver function and dynamic coagulation parameters is essential. These parameters should include PT/INR, fibrinogen, D-dimer, platelet count, and, when indicated, levels of specific procoagulant and anticoagulant factors, to concurrently identify impaired hepatic synthesis and consumptive coagulopathy. Second, in the presence of progressive coagulation factor consumption or evidence of DIC, management should not be limited to supportive replacement therapy (e.g., fresh frozen plasma) but should also target underlying procoagulant triggers. This includes controlling systemic inflammation, correcting contributors to endothelial injury, and, when appropriate, implementing adjunctive interventions such as plasma exchange or blood purification therapies.

### 4.4 Elevation of myocardial injury biomarkers

In the present study, four patients exhibited elevated myocardial injury biomarkers, such as cTnI, suggesting that acute 1,1-DCE poisoning may trigger a process of multi-organ injury. Elevation of myocardial biomarkers has been rarely reported in the existing toxicological literature. The observed increase in cTnI in several cases in this cohort indicates that the myocardium may also be subjected to either direct or indirect injury. Because electrocardiographic findings, echocardiographic data, and cardiac imaging were not systematically available, direct myocardial toxicity cannot be established from the present dataset. Potential mechanisms include potential direct cardiotoxic effects of reactive metabolites; secondary myocardial injury resulting from hepatic and renal dysfunction, metabolic acidosis, and hypoxia; as well as impaired myocardial perfusion due to systemic inflammation and microcirculatory disturbances (24–26). Although the current dataset does not allow for definitive differentiation among these mechanisms, the findings underscore the importance of incorporating myocardial enzyme monitoring into routine clinical assessment, to facilitate early detection and management of cardiovascular complications.

From a clinical management perspective, the mechanisms suggest that, in patients with suspected or confirmed acute 1,1-DCE poisoning, myocardial injury biomarkers should be incorporated into routine dynamic monitoring. In cases of progressive elevation of cTnI or concomitant electrocardiographic and echocardiographic abnormalities, a comprehensive evaluation encompassing ischemic, metabolic, and toxic etiologies is warranted, followed by targeted management. This includes correction of hypoxia, metabolic acidosis, and electrolyte disturbances, control of systemic inflammation, and appropriate cardiovascular support.

Furthermore, additional studies are needed to elucidate the direct effects of 1,1-DCE and its metabolites on cardiomyocyte mitochondrial function, programmed cell death pathways, and local inflammatory cell infiltration. Such investigations may provide a mechanistic basis for the rational application of targeted interventions, including antioxidant therapy, anti-inflammatory strategies, and membrane-stabilizing agents.

### 4.5 Clinical management implications

With regard to therapeutic and supportive strategies, existing evidence indicates that reactive metabolites of 1,1-DCE conjugate with GSH, leading to depletion of intracellular GSH reserves and suggesting a critical role of GSH homeostasis in determining toxic outcomes (27). Antioxidant therapies, such as N-acetylcysteine (NAC), have been shown in both animal and cellular studies to replenish intracellular GSH levels and enhance antioxidant defenses (28). Based on toxicological principles and prior experimental data, early administration of antioxidant support—including NAC—appears theoretically feasible and warrants systematic evaluation in future studies and clinical management guidelines. It should be noted, however, that the effects of NAC are not uniformly beneficial across different halogenated hydrocarbon toxicity models. Some animal studies suggest that drug–toxin interactions may be complex (29, 30). Therefore, its clinical application should be guided by experimental evidence and a cautious risk–benefit assessment.

Early initiation of treatment upon hospital admission may confer clinical benefits. In the present study, all patients received prompt, comprehensive management centered on organ support at an early stage of hospitalization, including respiratory support, renal replacement therapy, and component transfusion or plasma therapy for coagulation disorders. Six patients underwent plasma exchange during the disease course, primarily as a supportive intervention in the context of severe hepatic dysfunction and coagulopathy. Although blood purification strategies for critically ill patients—such as plasma exchange and CRRT—were frequently employed in this cohort and served as important life-sustaining measures, there is currently no evidence to suggest that routine extracorporeal therapies directly enhance the clearance of 1,1-DCE or its reactive intermediates. Their principal role is more likely supportive, targeting complications of organ failure induced by 1,1-DCE toxicity, including fluid management, correction of metabolic acidosis, renal function replacement, and adjunctive support in the setting of DIC or coagulation imbalance. Therefore, the potential role of more clearance-oriented techniques, such as high-flux adsorption or plasma exchange, requires further clarification through *in vivo* pharmacokinetic studies and well-designed clinical trials. Finally, this study proposes several practical and research-oriented recommendations. First, industrial settings should strengthen engineering controls and personal protective measures to prevent high-concentration, short-term exposures in confined spaces. Second, in clinical practice, patients with suspected high-dose exposure to halogenated hydrocarbons should undergo early implementation of a comprehensive multi-organ monitoring framework, including assessment of hepatic, renal, coagulation, myocardial, and inflammatory parameters, alongside timely consideration of blood purification and supportive strategies. Third, at the research level, priority should be given to the development of blood and urinary metabolite monitoring, as well as pharmacokinetic studies, to establish exposure–response relationships. In parallel, the true clinical benefits of antioxidant therapies (e.g., N-acetylcysteine) and extracorporeal clearance strategies should be systematically evaluated in both animal models and human studies. Fourth, the establishment of regional rapid-response systems and shared databases for occupational exposure incidents is recommended to facilitate evidence accumulation and improve emergency preparedness.

In summary, this study not only expands the empirical evidence on human intoxication with 1,1-DCE but also provides a practical framework to inform both clinical management and occupational health prevention strategies. Currently, no disease-specific clinical practice guidelines or expert consensus statements are available for the management of acute 1,1-DCE poisoning. Consequently, treatment strategies are largely extrapolated from general principles of toxicology, management of acute liver injury, acute kidney injury, and multiple organ dysfunction syndrome. Current management therefore remains predominantly supportive, focusing on early recognition of organ injury, close monitoring, timely organ support, and prevention of secondary complications.

### 4.6 Strengths and limitations

This study has several notable strengths. First, this case series originated from a single acute occupational exposure event, with a high degree of consistency in the exposure agent, route, and time window across patients. This substantially reduces the exposure heterogeneity and recall bias commonly encountered in previously reported sporadic cases, thereby enhancing the comparability of clinical manifestations and laboratory dynamics among individuals. Second, to our knowledge, this represents the largest reported case series of acute human intoxication with 1,1-DCE. The study systematically collected and dynamically analyzed multi-organ functional parameters—including hepatic, renal, coagulation, myocardial, and inflammatory markers—providing a more comprehensive characterization of the clinical toxicity spectrum compared with prior reports that were largely limited to single-organ involvement or single time-point observations. Third, all patients were managed at a single medical center, where clinical management protocols and laboratory testing methods were relatively standardized. This consistency helps minimize information bias arising from inter-institutional differences in diagnostic criteria or therapeutic strategies, thereby improving the internal validity of the study findings.

However, several limitations of this study should be acknowledged. First, this was a single-center retrospective case series with a limited sample size, which restricts the ability to perform robust statistical inference and subgroup analyses. Second, as all included patients were hospitalized following acute, high-dose exposure, the study population is inherently skewed toward more severe cases. Consequently, the findings may overestimate the severity of 1,1-DCE intoxication, and caution is warranted when extrapolating these results to individuals with mild or subclinical exposure. In addition, this study primarily focused on acute-phase manifestations during hospitalization and lacked systematic long-term follow-up data. Therefore, it remains unclear whether survivors may develop delayed hepatic or renal dysfunction, cardiovascular complications, or other chronic health effects. Future studies should incorporate multicenter collaborative designs, integrate toxicokinetic monitoring, and include long-term follow-up to better elucidate the dose–response relationship, key pathophysiological mechanisms, and optimal management strategies for acute 1,1-DCE poisoning.

## 5 Conclusion

This case series systematically characterizes the clinical features and multi-organ injury patterns in 13 patients with acute 1,1-DCE poisoning. The findings indicate that high-dose acute 1,1-DCE exposure can rapidly induce severe hepatic injury as the predominant manifestation, while also affecting renal function, the coagulation system, and the myocardium. These clinical manifestations align with previously described mechanisms of metabolic activation and oxidative stress, but present as more severe phenotypes in humans. The study underscores that 1,1-DCE intoxication should be considered a systemic toxic process, necessitating early, dynamic multi-organ monitoring and comprehensive supportive management in clinical practice. By expanding the empirical understanding of the human clinical spectrum of acute 1,1-DCE poisoning, this work provides a foundation for future mechanistic investigations and optimization of therapeutic strategies.

## Data Availability

The raw data supporting the conclusions of this article will be made available by the authors, without undue reservation.

## Glossary

1,1-DCE: 1,1-Dichloroethylene
ALT: Alanine aminotransferase
AST: Aspartate aminotransferase
AKI: Acute kidney injury
VV-ECMO: Veno-venous extracorporeal membrane oxygenation
CRRT: Continuous renal replacement therapy
PVDC: Polyvinylidene chloride
CYP2E1: Cytochrome P450 2E1
KDIGO: Kidney Disease: Improving Global Outcomes
ULN: Upper limit of normal
PT: Prothrombin time
APTT: Activated partial thromboplastin time
INR: International normalized ratio
cTnI: Cardiac troponin I
ICU: Intensive care unit
TBIL: Total bilirubin
PALB: Prealbumin
CKD: Chronic kidney disease
RBC: Red blood cell
WBC: White blood cel
GSH: Glutathione
DIC: Disseminated intravascular coagulation
TF: Tissue factor
TNF-*α*: Tumor necrosis factor-α
NAC: N-acetylcysteine
